# Cancer Care in the Gaza Strip Before and During War: Patient Experiences of Treatment Disruption, Unmet Support Needs, and Interdisciplinary Implications for Medicine, Nursing, and Social Work

**DOI:** 10.1002/pon.70575

**Published:** 2026-08-11

**Authors:** Baraka Abusafia, Yaser Snoubar, Zekiye Turan, Gürkan Sert, Feras Albess

**Affiliations:** ^1^ Department of Nursing Faculty of Health Sciences Sakarya University Serdivan Türkiye; ^2^ Social Sciences Department (Social Work Program) College of Arts and Sciences Qatar University Doha Qatar; ^3^ Department of Obstetric and Gynecological Nursing Faculty of Health Sciences Sakarya University Serdivan Türkiye; ^4^ Medical Faculty, Department of Medical History & Ethics Marmara University Istanbul Türkiye; ^5^ Department of Law Faculty of Administration and Law Al‐Aqsa University Gaza Palestine

**Keywords:** cancer care, gaza strip, healthcare access, psychosocial support, war

## Abstract

**Background:**

Cancer patients in the Gaza Strip have long faced major barriers to timely diagnosis, treatment, and continuity of care. These barriers have become substantially more severe under war conditions, displacement, and the collapse of health services. This study aimed to explore the experiences of adults with cancer in the Gaza Strip before and during war, with a particular focus on access to healthcare, support mechanisms, and basic living conditions.

**Methods and Results:**

This qualitative study was conducted with 12 adults diagnosed with cancer and living in shelters, displacement centres, or tents in the Gaza Strip. Data were collected through in‐depth semi‐structured interviews in Arabic and analysed using content analysis. The analysis identified six key themes describing cancer care in the Gaza Strip, organized within two temporal categories: three themes characterized the pre‐war period and three themes characterized the wartime period. The pre‐war themes were problems in access to healthcare services, support mechanisms, and positive aspects related to diagnosis and treatment. The wartime themes were problems in access to healthcare services, insufficiency of support mechanisms, and inadequacy of basic living conditions. Participants reported that even before the war, they experienced delayed diagnosis, fragmented treatment pathways, financial hardship, and limited psychosocial support, although some described positive experiences with functioning hospitals and treatment availability. During the war, participants described severe disruption of medical follow‐up, hospital inaccessibility, medication shortages, inability to travel for treatment, irregular or insufficient support, displacement, food insecurity, and worsening physical and psychological burden.

**Conclusions:**

The findings show that cancer care in the Gaza Strip was already fragile before the war and became profoundly disrupted during it. The experiences of patients highlight the need for integrated responses that address not only treatment continuity, but also psychosocial support, nutrition, and basic survival needs. The study also underscores the interdisciplinary relevance of cancer care in conflict settings for medicine, nursing, and social work. By showing how treatment uncertainty, disrupted support, and unmet survival needs interact, the study identifies patient‐level psycho‐oncological priorities for cancer care in conflict and crisis settings.

## 1 Background

The Palestinian health system has long operated under conditions of fragmentation, underfunding, limited governance capacity, and weak evidence‐informed policy development [1]. In the Gaza Strip, prolonged isolation has had devastating effects on health services, living conditions, population health, and service capacity, contributing to adverse health outcomes [2]. These structural constraints have had particularly serious consequences for patients with cancer, who often require timely diagnosis, referral, and access to specialized treatment.

Access to essential health services for Palestinians in the West Bank and Gaza Strip remains severely restricted, especially for patients with cancer who need referral pathways for diagnosis and treatment. In the Gaza Strip, cancer patients may wait for months before receiving treatment, and the process of obtaining permits to seek medical care outside the Gaza Strip is often lengthy, uncertain, and unsuccessful for many patients [3]. In addition, the resources available in the Gaza Strip hospitals are insufficient for both cancer diagnosis and treatment. Critical components of the cancer care continuum, including nuclear medicine imaging for cancer staging, radiotherapy services, and several interventional procedures, remain unavailable [3]. According to available evidence, cancer is the second leading cause of disease‐related death in Palestine and accounts for 12.4% of all deaths. Because many cases are detected at a late stage, treatment becomes more difficult, survival outcomes worsen, and quality of life is negatively affected [4].

Previous research has shown that cancer patients in the Gaza Strip were already living under major psychological, social, and financial strain before the current war. A 2017 study of 380 cancer patients in the Gaza Strip found that siege‐related stressors, including high prices, restricted movement, work disruption, and shortages of electricity, gas, and fuel, were strongly associated with anxiety and depression. The authors recommended the development of specialized psychological support services for cancer patients [5]. Another 2017 study, based on 14 in‐depth interviews and 364 cross‐sectional questionnaires administered in Al‐Shifa and the European the Gaza Strip Hospital, reported that adult cancer patients expressed substantial unmet needs related to emotional support, financial support, stigma reduction, recreational opportunities, better healthcare services, and protection of their rights. The same study also found low quality‐of‐life scores across multiple domains [4].

Social support is a central component of the illness experience, particularly in life‐threatening conditions such as cancer, because it can reduce emotional suffering, loneliness, and distress, while promoting overall health and well‐being [6, 7]. In practical terms, support may help patients obtain information, access resources, reduce stress, and improve both physical and psychological adjustment. Previous evidence also suggests that partner support is positively associated with quality of life among women with breast cancer [8].

In the Gaza Strip, women with breast cancer face additional disadvantages linked to structural inequalities shaped by the political context. These include delayed diagnosis, limited treatment pathways, and uncertainty regarding access to treatment outside the Gaza Strip. Research has also shown that ambiguity surrounding treatment plans and treatment side effects increases the importance of social support networks in patients' lives [7]. Evidence further suggests that living in environments characterized by armed conflict and social instability increases the risk of depression and psychological distress, which may in turn worsen cancer outcomes [9]. In this context, Abu‐Odah et al. [10] found that patients with advanced cancer in the Gaza Strip reported high levels of psychological distress, anxiety, and depression compared with patients with advanced cancer in other settings.

Another study focussing on women with breast cancer in the Gaza Strip examined the ways in which sociocultural factors shape social support among survivors. The findings indicated that illness experiences and support levels were influenced by three interrelated factors: marriage‐centred social expectations, normative meanings attached to illness, and women's social identities. The study recommended group‐specific support interventions, greater public awareness within patients' social networks, and financial empowerment strategies to reduce the broader social burden of cancer [7].

Since the war that began on 7 October 2023, civilian suffering and destruction in the Gaza Strip have reached unprecedented levels. Large numbers of families have been displaced to overcrowded camps characterized by insecurity, violence, and scarcity of resources. Healthcare services have been severely affected by damage to hospitals and healthcare facilities, while the remaining services have been overwhelmed by the large number of wounded civilians. This has substantially reduced access to healthcare and contributed to a worsening public health crisis [11, 12]. Recent WHO data further illustrate the scale of this disruption. As of 31 March 2026, all 36 hospitals in the Gaza Strip and most primary healthcare centres had been damaged; only 19 of 36 hospitals (53%) were partially functional, and 108 of 209 primary healthcare facilities (52%) were operational. The same WHO situation update reported that 937 attacks on health care had been recorded as of 31 January 2026, with 993 people killed and 1654 injured in those attacks, 176 health facilities affected, 36 hospitals damaged, and 216 ambulances affected [13].

At the same time, severe shortages of food, clean water, sanitation, and shelter have further undermined health and survival. Many families are living with insufficient food, unsafe water, poor hygiene conditions, and limited access to toilets and bathing facilities, all of which increase the risk of illness and deepen suffering [11, 12]. The economic situation has also deteriorated sharply. Local businesses, employment opportunities, and income sources have been disrupted, resulting in rising unemployment, poverty, fear, and daily uncertainty [14]. For cancer patients and their families, the financial burden of treatment, transportation, medications, and daily care becomes even more severe in times of war, while displacement may weaken or completely disrupt existing support systems [15].

Restrictions on supplies, combined with direct damage to health infrastructure, have further aggravated the challenges faced by cancer patients in the Gaza Strip. Access to chemotherapy, radiotherapy, diagnostic services, and supportive care has become even more limited [15]. Before 7 October 2023, cancer was already among the leading causes of death in Palestine, with 2047 new cancer cases reported in 2022 [11]. Since the war, cancer patients in the Gaza Strip have faced intensified stressors, including lack of access to radiotherapy, systemic treatment, and adequate nutrition. Approximately 2000 oncology patients requiring active treatment have been placed at serious risk, and reports have indicated that thousands of women with cancer are in urgent need of medical attention while services have been largely interrupted [11, 15].

The psychological condition of cancer patients is especially vulnerable to deterioration in conflict settings. Ongoing violence, displacement, fear, insecurity, bereavement, and uncertainty may intensify distress during an already demanding period of diagnosis and treatment, potentially affecting recovery, coping, and quality of life [15].

Against this background, there remains a need for qualitative evidence on how adults with cancer in the Gaza Strip experience healthcare access, support mechanisms, and basic living conditions before and during war. Such evidence is important not only for understanding treatment disruption in conflict settings, but also for informing interdisciplinary responses across medicine, nursing, and social work. Thus, this study aimed to examine the lived experiences of adults with cancer in the Gaza Strip before and during the war, with a focus on healthcare access, support mechanisms, and basic living conditions. It further explored how war‐related disruption, displacement, and the collapse of services affected continuity of cancer care and patients' physical, psychological, and social well‐being. To maintain a clear qualitative focus, the study was guided by two broad research questions:

1. How do adult cancer patients in the Gaza Strip experience the continuum of healthcare access, support mechanisms, and basic living conditions before and during the war?
2. How do war‐related disruption and displacement shape continuity of cancer care and patients' physical, psychological, and social well‐being?

## 2 Materials and Methods

### 2.1 Study Design

This study employed a qualitative descriptive design to explore how adults living with cancer in the Gaza Strip experienced cancer care before and during the war, with particular attention to healthcare access, support mechanisms, basic living conditions, and psycho‐oncological needs. This design was considered appropriate because the study aimed to provide a direct, context‐sensitive account of participants' experiences and to identify practice‐relevant care needs in an under‐researched conflict setting, rather than to generate a formal theory or compare predefined diagnostic subgroups. The reporting of this qualitative interview study was guided by the Consolidated Criteria for Reporting Qualitative Research (COREQ), a 32‐item checklist for interviews and focus groups [16]. A completed COREQ checklist is provided as Supporting Information S1.

### 2.2 Setting and Participants

The study was conducted with 12 adult cancer patients living in shelters, displacement centres, or tents in the Gaza Strip at the time of data collection. Participants were recruited using purposive sampling. Eligible participants were adult men and women who had been diagnosed with cancer and required ongoing cancer treatment or follow‐up. Participation was voluntary. Individual in‐depth interviews were conducted with each participant, and each interview lasted approximately 45–60 minutes. With participants' consent, the interviews were audio‐recorded. To ensure confidentiality, each participant was assigned a unique identifier (e.g., P1, P2). Data collection continued until sufficient depth had been reached to address the study questions.

The socio‐spatial context of data collection was highly constrained. Participants were living in non‐institutional displacement settings, including overcrowded shelters, displacement centres, and tent clusters where privacy, transport, electricity, food, clean water, sanitation, and access to health facilities were limited. Interviews were arranged at times and places judged as safe and feasible by the local field researcher and participants, with attention to minimizing distress and protecting confidentiality within these difficult conditions.

The timing of cancer diagnosis was recorded to contextualize participants' narratives but was not used as an exclusion criterion. Adults diagnosed either before or during the war were eligible if they required ongoing cancer treatment or follow‐up and were living under displacement conditions at the time of data collection. This inclusive approach was chosen because the study aimed to examine disruption across the cancer‐care continuum rather than to compare disease‐specific or diagnosis‐timing‐specific subgroups. Eligibility was also not restricted to a single cancer type. The sample included several diagnoses, including breast, colon/colorectal, and thyroid cancers, which are among commonly reported cancers in the Gaza Strip, while it did not include all common cancer types, such as lung cancer [17]. Disease heterogeneity was therefore retained deliberately, and the findings are interpreted as cross‐cutting experiences of treatment disruption, support needs, and living conditions rather than as cancer‐type‐specific trajectories. Given the composition of the purposive sample, the study was not designed to compare experiences by sex or to examine gender‐related differences; therefore, no male‐specific or sex‐comparative conclusions are drawn. Similarly, the cross‐cutting themes should not be interpreted as indicating that experiences, needs, or treatment disruptions were equivalent across different cancer types or treatment trajectories.

### 2.3 Data Collection and Interview Guide

Data were collected between 12 December 2024 and 20 March 2025 using a semi‐structured interview guide. The guide included questions on participants' socio‐demographic characteristics, including age, marital status, educational level, place of residence, duration of stay in shelters, cancer diagnosis, and time since diagnosis. The interview guide explored participants' experiences of cancer treatment before the war and during the current war. The interviews focussed on experiences related to diagnosis, treatment, and follow‐up care, the impact of medical care on daily life, financial burden, available family, institutional, and psychosocial support, emotional and psychological well‐being, coping strategies, and the urgent needs of cancer patients under conditions of war and displacement. The interview process remained flexible, allowing probing and follow‐up questions in response to participants' narratives. At the end of each interview, participants were invited to share any additional comments they considered important. In line with the two overarching research questions, the interview guide used more specific prompts to explore: access to diagnosis, treatment, and follow‐up before the war; changes in healthcare access during the war; forms of family, institutional, and psychosocial support; the impact of war and displacement on daily living conditions; and the physical and psychological consequences of treatment disruption and deteriorating living conditions.

Because the study examined the lived experiences of adults with cancer under active war and displacement conditions, fieldwork was conducted in the Gaza Strip by the local field researcher. He facilitated access to participants in shelters, displacement centres, and tent settings, arranged interviews according to safety and feasibility, and provided contextual clarification during analysis. The study was community‐based and conducted in non‐institutional displacement settings rather than within a single hospital or formal care institution.

Specific ethical approval details, consent procedures, and confidentiality protections are reported in Section 2.6 (Ethical considerations). Author roles were clarified as follows. Feras Albess, the Gaza Strip‐based local field researcher, conducted the interviews, facilitated community access, and provided contextual clarification during interpretation. Baraka Abusafia contributed to the initial review of the interview materials and the development of preliminary codes and categories. Yaser Snoubar led the methodological refinement and analytic supervision of the study, independently reviewed the interview materials, contributed substantially to coding, category organization, and theme development, and guided the interpretation of findings from social work and interdisciplinary practice perspectives. Zekiye Turan contributed to interpretation from a nursing and cancer‐care perspective, and Gürkan Sert contributed to ethical and contextual interpretation. All authors reviewed the developing coding structure, discussed the final themes, and approved the interpretation presented in the manuscript.

### 2.4 Data Analysis

The interview data were analysed using inductive qualitative content analysis, guided by the preparation, organization, and reporting phases described by Elo and Kyngäs [18]. Analysis began after completion of the interviews and proceeded iteratively through repeated engagement with the interview materials. First, the audio‐recorded interviews and interview materials were reviewed carefully to achieve immersion and identify meaning units related to healthcare access, treatment disruption, support mechanisms, basic living conditions, and psychosocial burden. Open codes were then generated from participants' accounts, compared across pre‐war and wartime narratives, grouped into categories, and abstracted into six analytical themes organized within two temporal categories. To strengthen coding rigour, Baraka Abusafia and Yaser Snoubar independently reviewed the interview materials and preliminary codes. Differences in coding, category grouping, or theme naming were discussed among the research team and resolved by consensus, with the local field researcher consulted when clarification of local expressions, treatment pathways, or displacement context was needed. Table 1 provides an illustrative example of how meaning units were coded, grouped, and abstracted into analytical themes.

**TABLE 1:** Illustrative coding tree from meaning units to analytical themes.

Condensed meaning unit	Open code	Category	Analytical theme	Temporal category
Participant described delayed surgery, referral paperwork, and travel outside the gaza strip for unavailable treatment.	Delayed referral and treatment outside the gaza strip	Fragmented treatment pathway	Problems in access to healthcare services	Pre‐war period
Participant reported stopping treatment because reaching hospital became unsafe and difficult during the war.	Treatment interruption due to unsafe access	Interrupted follow‐up and treatment continuity	Problems in access to healthcare services	Wartime period
Participant reported irregular food or financial assistance and unmet basic needs.	Irregular humanitarian support	Insufficient formal support	Insufficiency of support mechanisms	Wartime period
Participant described tent life, physical exhaustion, unaffordable healthy food, and lack of income.	Displacement‐related hardship	Deteriorated living and nutrition conditions	Inadequacy of basic living conditions	Wartime period

### 2.5 Trustworthiness

Several strategies were used to enhance trustworthiness and to align reporting with relevant COREQ domains, including research team and reflexivity, study design, and analysis and reporting [16]. Credibility was supported through expert review of the interview guide, repeated engagement with participants' accounts, the use of direct quotations, and team‐based discussion of codes, categories, and themes. Dependability was strengthened through independent review of interview materials by more than one author and consensus‐based resolution of coding disagreements. Confirmability was supported by retaining a clear link between meaning units, codes, categories, and themes, and by involving the local field researcher in clarifying contextual meanings while the wider team critically reviewed interpretation.

Researcher reflexivity was addressed explicitly. The local field researcher was himself living in the Gaza Strip under war conditions, which created both contextual proximity and a potential risk of emotional burden or shared‐assumption bias. The research team therefore treated this positionality as an analytic consideration during interpretation. The field researcher did not occupy a treatment‐provider role in relation to participants. Rapport was established through culturally familiar communication, clear explanation of the study purpose, reassurance about confidentiality and voluntariness, and allowing participants to decide whether and how much they wished to disclose.

Member checking of transcripts or final themes was not conducted because repeated contact with displaced participants during active conflict was unsafe, logistically uncertain, and could have increased participant burden. The analysis therefore relied on careful use of participant quotations, team‐based coding review, and contextual clarification by the field researcher. Formal data triangulation with additional qualitative sources was not undertaken; however, interpretation was contextualized using field‐level clarification and the humanitarian and health‐system reports cited in the manuscript. Thick description of shelters, displacement centres, and tent settings was added to the Methods section to support transferability.

### 2.6 Ethical Considerations

Ethical approval for this study was obtained from the Social and Human Sciences Ethics Committee of Sakarya University, Türkiye (approval/reference no. E‐61923333‐050.99‐428786; meeting no. 79; decision no. 25; approved on 11 December 2024). Prior to participation, all participants were informed about the purpose of the study, the voluntary nature of their involvement, their right to refuse participation or withdraw at any time without penalty, and the measures taken to protect confidentiality. Verbal informed consent was obtained from all participants before the interviews and audio‐recording. Written consent was not sought because the study was conducted under wartime displacement conditions in shelters, displacement centres, and tents, where obtaining signed consent forms was not always feasible and could have increased participants' discomfort or concern. Participation proceeded only after each participant had clearly agreed to take part. To protect privacy and confidentiality, all identifying information was removed from the interview material, and pseudonymous codes (e.g., P1, P2) were used in reporting the findings. No personally identifiable information is reported in this manuscript. To further protect anonymity, individual demographic and clinical characteristics were presented in aggregated form, and potentially identifying details were minimized in the quotations. Therefore, separate consent for publication of identifiable individual details was not applicable.

## 3 Results

### 3.1 Participant Characteristics

The study included 12 adults living with cancer in the Gaza Strip. As detailed in Table 2, the sample was predominantly female (11 women and one man), ranged in age from 30 to 72 years, and included participants with several cancer diagnoses. Most participants were married, and educational backgrounds ranged from primary school to bachelor's degree level. To protect confidentiality in this small and highly vulnerable sample, participant information is presented in a de‐identified format using participant codes, age ranges, and grouped numbers of children rather than exact potentially identifying details.

**TABLE 2:** De‐identified socio‐demographic and clinical characteristics of participants.

Participant	Sex	Age range	Educational status	Marital status	Number of children (grouped)	Cancer diagnosis
P1	Female	30–39	High school	Married	1–3	Thyroid cancer
P2	Female	30–39	Secondary school	Married	7+	Breast cancer
P3	Female	30–39	High school	Married	4–6	Brain tumour
P4	Female	30–39	High school	Married	4–6	Brain tumour
P5	Female	40–49	Diploma	Married	4–6	Breast cancer
P6	Male	40–49	High school	Married	1–3	Testicular cancer
P7	Female	50–59	Bachelor's degree	Married	1–3	Breast cancer
P8	Female	60+	Primary school	Single	Not reported	Colon cancer
P9	Female	50–59	High school	Married	7+	Breast cancer
P10	Female	40–49	Primary school	Married	7+	Thyroid cancer
P11	Female	50–59	High school	Married	1–3	Breast and ovarian cancer
P12	Female	60+	Primary school	Married	4–6	Breast cancer

The participant group included people whose cancer trajectories began before the war as well as participants who described new or evolving diagnostic and treatment needs during the war. Because several accounts connected pre‐war treatment histories with wartime disruption, participants were analysed together to identify shared patterns across access to care, support, and living conditions.

### 3.2 Overview of Themes

**FIGURE 1:**
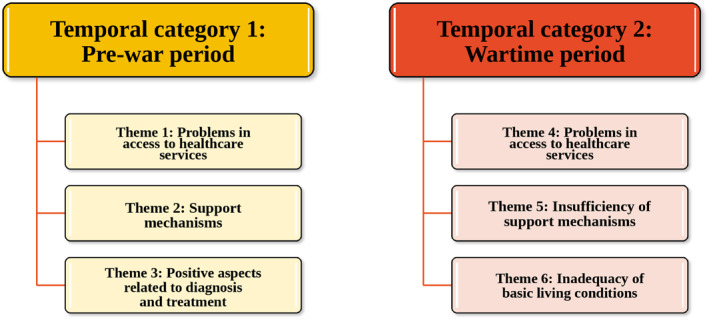
Temporal categories and six analytical themes.

Content analysis showed that participants described their experiences in relation to two broad periods: before the war and during the war. Across both periods, cancer care was shaped by substantial barriers, although these challenges became markedly more severe during the war. Our analysis identified six key themes describing cancer care in the Gaza Strip, organized within two temporal categories. Three themes characterized the pre‐war period: problems in access to healthcare services, support mechanisms, and positive aspects related to diagnosis and treatment. Three themes characterized the wartime period: problems in access to healthcare services, insufficiency of support mechanisms, and inadequacy of basic living conditions (Figure 1).

### 3.3 Pre‐War Period

Participants described the pre‐war period as characterized by substantial difficulties in access to healthcare services and limited support mechanisms, although some also reported positive experiences related to diagnosis and treatment. Within this temporal category, three analytical themes emerged: problems in access to healthcare services, support mechanisms, and positive aspects related to diagnosis and treatment.

#### 3.3.1 Problems in Access to Healthcare Services

Before the war, participants described cancer care as involving considerable logistical, procedural, and financial challenges. These included delays in diagnosis and treatment, complex referral and travel procedures, postponement of surgeries due to limited hospital resources, the need to travel outside the Gaza Strip for specific forms of treatment, and the financial burden associated with care.

A woman in her 30s with thyroid cancer described a prolonged referral process and the need to seek radioactive iodine treatment outside the Gaza Strip: ‘My treatment was a struggle even before the war. I was diagnosed in January 2019 during the COVID‐19 pandemic and waited until May to have surgery. After that, I spent a long time following up on the medical referral procedures, completing the necessary paperwork, and arranging and coordinating travel to Egypt for treatment there, as radioactive iodine was not available in the Gaza Strip.’ (P1).

A woman in her 30s with a brain tumour described hospital movement, surgical delay, and worsening symptoms: ‘I faced many difficulties, such as moving between hospitals for examinations and the long wait before the operation. Then I was discharged from the hospital without the operation, which increased my suffering due to the severe symptoms I had, such as severe headaches and loss of consciousness.’ (P4).

A woman in her 50s with breast cancer described the diagnostic period as psychologically stressful: ‘The diagnosis period was very difficult and psychologically stressful. At first, I underwent intensive examinations at Al‐Rantisi Hospital over three consecutive months, which included imaging and comprehensive blood tests, including an immune test.’ (P7).

A woman in her 50s with breast cancer described how treatment was fragmented across several locations: ‘I travelled to Jerusalem outside the Gaza Strip to receive radiation treatment, then returned to the Gaza Strip where I received six doses of chemotherapy. As for the surgery to remove the lump, I travelled to Egypt and underwent the operation there. Thus, my treatment journey was distributed among several places, as each stage of treatment took place in a different country or city.’ (P9).

A woman in her 50s with breast and ovarian cancer highlighted the financial burden of treatment before the war: ‘The economic situation was rather bad at that time, as we had to bear the expenses of going to the hospital, in addition to the cost of some treatments that we had to buy at our own expense.’ (P11).

#### 3.3.2 Support Mechanisms

Participants indicated that, even before the war, social, economic, and psychological support was often inadequate. The diagnosis and treatment process was described as a major source of shock, stress, and anxiety, and cancer was often perceived as a life‐threatening condition associated with fear. During this period, support was derived mainly from family members, including spouses, children, parents, and siblings. Some participants also reported receiving psychological support from organizations and associations.

A woman in her 40s with thyroid cancer described relying on her husband and children for living and treatment expenses: ‘During my treatment, I received great support from my children and my husband, who worked in the West Bank and regularly sent me living and treatment expenses.’ (P10).

A woman aged 60 or older with colon cancer described support from siblings and their families: ‘My brothers and their wives are my support in every way, giving me support and assistance in any way they can.’ (P8).

A man in his 40s with testicular cancer emphasized his wife's psychological support during treatment: ‘Throughout the treatment period, I suffered from the side effects of chemotherapy and cancer medications, which increased my suffering. But despite all of that, my wife was always by my side, providing me with psychological support and helping me endure.’ (P6).

A woman in her 30s with breast cancer described a lack of family and social support: ‘As for family or social support, I completely lacked it, whether from my family or my husband. Since the beginning of the diagnosis until now, I have not received any family or social support.’ (P2).

A woman in her 50s with breast and ovarian cancer described institutional psychological support before the war: ‘I received support from some associations and institutions that provided me with psychological support during that period, as they organized recreational days and trips for us to support us psychologically. All of this had a great positive impact on my psychological state.’ (P11).

#### 3.3.3 Positive Aspects Related to Diagnosis and Treatment

Although the pre‐war period was largely marked by difficulties, some participants also reported positive experiences related to diagnosis and treatment. These included access to functioning hospitals, regular medical follow‐up, availability of medications, and financial coverage through health insurance.

A woman in her 40s with breast cancer described regular follow‐up at the Turkish Friendship Hospital before the war: ‘I was receiving treatment at the Turkish Friendship Hospital, where I had monthly follow‐ups with the doctor. Things were going well, and I was receiving medical care at the hospital without any problems.’ (P5).

A man in his 40s with testicular cancer recalled being able to travel for treatment before the war: ‘In 2019, I was diagnosed with a tumour in one of my testicles, had it removed, and underwent chemotherapy. I was able to travel to the West Bank and Jerusalem, where I received treatment.’ (P6).

A woman in her 50s with breast and ovarian cancer compared pre‐war service availability with the wartime situation: ‘Before the war, cancer treatment was more available than it is now. Hospitals were functioning efficiently, such as Al‐Rantisi Hospital and the Turkish Hospital in the Gaza Strip. Roads were passable, transportation was available, and treatment was available.’ (P11).

A woman aged 60 or older with breast cancer described medication availability and insurance coverage before the war: ‘I received treatment at the Turkish Friendship Hospital in the Gaza Strip, where medications were available and covered by health insurance. The costs of the surgery and treatment in the hospital were fully covered by health insurance. Before the war, things were good in terms of the availability of treatment and medication.’ (P12).

### 3.4 Wartime Period

Participants consistently reported that the onset of war intensified pre‐existing challenges and created new barriers across multiple domains of life. Within the wartime temporal category, three analytical themes emerged: problems in access to healthcare services, insufficiency of support mechanisms, and inadequacy of basic living conditions.

#### 3.4.1 Problems in Access to Healthcare Services

Participants emphasized that, during the war, access to hospitals became extremely difficult. Many facilities were destroyed, closed, or functioning only partially, and regular medical follow‐up was often interrupted. The complete disruption of travel outside the Gaza Strip further limited access to specialized treatment. Medications were reported to be scarce or unavailable, their prices had increased, and some participants were forced to discontinue treatment. Participants also described delays in diagnosis and difficulties in obtaining further investigation or surgical care.

A woman in her 30s with thyroid cancer explained how displacement and family disruption made hospital access more difficult: ‘After we were displaced to the camp and my husband was arrested, the hospital became far away from us, and going to it requires time and transportation, which is exhausting and expensive.’ (P1).

A woman in her 30s with a brain tumour described diagnostic uncertainty and the difficulty of referral during the war: ‘I was diagnosed in a hospital operating under difficult conditions, where it turned out that I had a new brain tumour. The tumour was diagnosed during the war, and the doctors said that it must be determined whether the tumour is benign or malignant. Until now, I need more tests and diagnoses to confirm the type of tumour. If it is diagnosed as malignant, I will have to look for a referral outside the Gaza Strip, which is a process that requires a lot of time and effort to obtain.’ (P4).

A woman in her 40s with breast cancer described stopping treatment because reaching hospital had become too difficult: ‘Because of the conditions we are going through due to the war, everything has become difficult, including reaching the hospital. I had to stop my treatment because going to the hospital has become very difficult.’ (P5).

A man in his 40s with testicular cancer described how closure of crossings prevented treatment outside the Gaza Strip: ‘After that, they prepared the papers for my transfer for treatment outside the Gaza Strip, due to the discovery of another tumour in my testicle. However, under these conditions, I cannot travel, as the crossings are closed and everything is at a standstill.’ (P6).

A woman aged 60 or older with colon cancer described shortages of required medical supplies and their effect on eating and drinking: ‘My nephew has to go to the hospital despite the difficulty and dangers of the road, and when he arrives, he gets a very small amount, not exceeding five to ten bags, which is not enough for my needs. Sometimes I have to reduce my food and drink intake so that I do not consume the bags quickly, and this has greatly affected my psychological and health condition.’ (P8).

A woman in her 40s with thyroid cancer described displacement and the danger of reaching health centres: ‘With the beginning of the war, our lives changed completely. We were displaced from our homes and moved to camps, where health centres were either destroyed or only operating to receive the wounded due to the large number of casualties resulting from the war and events. Everything in our lives changed, even reaching health centres or medical points became difficult and dangerous.’ (P10).

A woman in her 50s with breast and ovarian cancer described the inability to undergo surgery because resources were unavailable: ‘When I went to the hospital, the doctor told me that there are currently no possibilities for performing the operation because of its danger, as it may require removing part of the intestine and colon if the cancer cells spread. The doctor advised me to be patient at this stage, because performing the operation is not possible in the current situation, as the necessary capabilities are not available.’ (P11).

A woman aged 60 or older with breast cancer described being unable to reach hospital after displacement: ‘After we were displaced to this area, I am no longer able to go to the hospital. The situation is dangerous, and it is not possible to move to receive treatment in light of the war and the conditions we are living in.’ (P12).

#### 3.4.2 Insufficiency of Support Mechanisms

Participants stated that family members continued to provide emotional support during the war, but financial support became more limited. Support provided by organizations and associations, including financial aid, food assistance, and psychological support, was described as irregular and insufficient. Several participants expressed a clear need for more continuous and individualized support.

A woman in her 30s with thyroid cancer emphasized that emotional support alone was insufficient under survival‐threatening conditions: ‘Although psychological and emotional support is important, it is not enough to deal with the difficult circumstances we are living in. We are facing great difficulties, and we spend our whole day thinking about how to survive this war.’ (P1).

A woman in her 30s with breast cancer described lacking social, material, and emotional support after family disruption during the war: ‘I lack support in all its forms, especially social, material, and emotional support. I live alone with my children, as my husband was captured during the war and detained for four months. In addition, he does not provide me with any psychological, social, or emotional support.’ (P2).

A woman in her 30s with a brain tumour described continued moral support from close family members: ‘My mother, father, brothers, and husband are constantly trying to help me and support me morally.’ (P3).

A man in his 40s with testicular cancer described irregular assistance from associations: ‘The situation has become very dangerous, and although there are some associations that provide assistance to cancer patients like us, whether food or financial, this assistance is irregular and not continuous. When it is available, it is limited and does not fully cover our needs. However, we have no choice but to be patient in these harsh conditions.’ (P6).

A woman in her 40s with thyroid cancer described reliance on her children and the need for more continuous psychological support: ‘Mentally and emotionally, my children are my biggest supporters. As a cancer patient, I need more psychological support. It is true that some girls from an organization have provided me with psychological support sometimes, but I feel that this support is not enough. I need more continuous psychological support.’ (P10).

A woman in her 50s with breast and ovarian cancer summarized the insufficiency of available support: ‘We receive some support, but it is not enough to meet our basic needs.’ (P11).

#### 3.4.3 Inadequacy of Basic Living Conditions

Participants described a severe deterioration in living conditions during the war. They reported being unable to access adequate and healthy nutrition because of rising prices, food shortages, and insufficient aid. Essential foods such as vegetables, fruits, meat, eggs, and milk were often unavailable or unaffordable. Participants also described insecurity, danger, displacement, primitive living arrangements, unemployment, and financial hardship as central features of daily life.

A woman in her 30s with breast cancer described the physical and psychological exhaustion of daily life in tents: ‘Life in the tents has become very difficult, and everything in it is primitive. I wash clothes by hand, wash dishes, and take care of household affairs, all of which causes me severe pain and physical and psychological exhaustion.’ (P2).

A woman in her 30s with a brain tumour linked rising prices and lack of work to inadequate nutrition: ‘Prices have become very high, and I am unable to buy the food necessary for proper nutrition because of the high prices or the shortage of some foods in the markets. Also, job opportunities are non‐existent, and we are in dire need of financial support to be able to buy what we need as cancer patients.’ (P4).

A woman in her 40s with breast cancer emphasized the absence of adequate protein‐rich foods needed for her condition: ‘The conditions we live in are very difficult, and as a cancer patient I need continuous treatment. I also need proper nutrition. There is no healthy, complete diet, no eggs, meat, or fish, although I desperately need them to support my health.’ (P5).

A man in his 40s with testicular cancer described increased family responsibilities and financial burden: ‘My brother left behind three children, which increased the financial burdens and responsibilities on me, in light of the lack of job opportunities and any source of income.’ (P6).

A woman in her 50s with breast cancer described the gap between medical advice on nutrition and the reality of food unaffordability: ‘Doctors teach us about the importance of proper nutrition that is appropriate for our condition, but under the current circumstances, we cannot buy vegetables, fruits, and food items because their prices have risen significantly, and sometimes it is difficult to obtain them due to their shortage in the markets.’ (P7).

A woman aged 60 or older with colon cancer described the combined need for food, medication, medical supplies, clothing, and support: ‘As a cancer patient, under these difficult circumstances, I need healthy food suitable for my condition, in addition to the necessary medicine and medical supplies, as well as clothing and support in all its forms.’ (P8).

A woman in her 40s with thyroid cancer described the loss of job opportunities and worsening economic conditions: ‘The economic conditions became more difficult, and there were no more job opportunities available, which made matters worse and made the situation more complicated.’ (P10).

## 4 Discussion

The present study explored the experiences of adults living with cancer in the Gaza Strip before and during the war, with a particular focus on healthcare access, support mechanisms, and basic living conditions. The findings indicate that cancer care in the Gaza Strip was already characterized by substantial structural, financial, and psychosocial barriers before the war, and that these challenges intensified dramatically with the onset of war and displacement. Overall, the findings point to a continuum of vulnerability in which pre‐existing weaknesses in cancer care were transformed into a severe humanitarian and healthcare crisis.

### 4.1 Fragile Cancer Care Before the War

The findings show that, even before the war, cancer patients in the Gaza Strip faced substantial and multifaceted barriers to accessing care. Participants described fragmented care pathways marked by delays in diagnosis and treatment, complex referral and administrative procedures, the need to travel outside the Gaza Strip for specialized services, and the financial burden associated with treatment. Surgical postponements due to limited hospital capacity and resource shortages further compounded these difficulties. These findings are consistent with previous studies from the Gaza Strip and other conflict‐affected settings, which have documented how referral barriers, administrative restrictions, and shortages in specialized services delay cancer diagnosis and treatment and compromise continuity of care [19, 20, 21, 22]. They also support evidence suggesting that fragile health systems and resource limitations are major barriers to effective cancer care in low‐resource and conflict‐affected contexts [23].

The study also highlights the central but uneven role of support mechanisms in the pre‐war period. Participants described a heavy reliance on informal support systems, particularly family members, for emotional, psychological, and financial support. However, this support was not consistently available, and some participants reported a complete absence of family or social support. At the same time, access to formal psychosocial or institutional support was limited. These findings align with previous literature showing that cancer patients in vulnerable settings often depend primarily on family networks when structured psychosocial services are weak or unavailable [20]. From a theoretical perspective, Social Support Theory suggests that supportive social relationships can buffer the harmful effects of stress and psychological distress [24]. Empirical evidence similarly indicates that strong social support is associated with better treatment adherence, improved psychological well‐being, and, in some contexts, improved survival among cancer patients [25]. In contrast, limited access to psychosocial care may intensify uncertainty, fear, and distress, especially when cancer is perceived as a life‐threatening condition [26].

At the same time, participants did not describe the pre‐war period as uniformly negative. Some reported positive experiences related to diagnosis and treatment, including access to functioning hospitals, regular follow‐up, available medications, and partial or full treatment coverage through health insurance. These accounts suggest that, despite severe constraints, some elements of cancer care remained functional before the war. Similar observations have been reported in other low‐resource settings, where partial service functionality and supportive healthcare encounters may mitigate some of the burden associated with chronic illness and treatment [27, 28, 29]. Taken together, these findings suggest that the pre‐war cancer care system in the Gaza Strip was already under considerable strain, but had not yet fully collapsed. This pre‐existing fragility likely reduced the resilience of the system and intensified the effect of subsequent war‐related disruption.

### 4.2 Escalation of Vulnerability and Disruption of Care During the War

The findings further demonstrate that the onset of war dramatically worsened pre‐existing barriers and resulted in a near‐collapse of cancer care. Participants described severe disruption in access to healthcare, including destruction or closure of hospitals, interruption of regular follow‐up, inability to travel for treatment outside the Gaza Strip, medication shortages, rising treatment‐related costs, and the suspension or delay of necessary interventions. These findings are consistent with growing evidence from conflict‐affected settings showing that war severely disrupts oncology services, delays diagnosis, interrupts treatment, and increases the risk of adverse health outcomes [30, 31, 32, 33]. In this sense, the war did not create a new set of challenges in isolation; rather, it intensified and compounded longstanding weaknesses in the cancer care system.

The findings also underscore the deterioration of support mechanisms during the war. Although family members remained an important source of emotional support, their capacity to provide financial and practical assistance was significantly reduced under conditions of displacement, poverty, insecurity, and family separation. Support provided by organizations and associations was described as irregular, fragmented, and insufficient, particularly in relation to food aid, financial support, and psychological care. Participants emphasized the need for more continuous and individualized psychosocial support. These findings are consistent with previous studies indicating that humanitarian crises often weaken both formal and informal support systems, while increasing the need for sustained psychosocial care [34, 35, 36, 37]. The findings therefore suggest that psychosocial support in conflict settings should not be treated as secondary to medical care, but rather as an integral component of cancer care.

Another important finding concerns the marked deterioration in basic living conditions. Participants described food insecurity, poor shelter conditions, unemployment, insecurity, and severe financial hardship as central features of daily life during the war. For patients with cancer, these conditions were not merely background stressors; they directly affected physical strength, treatment tolerance, emotional well‐being, and the ability to manage illness. Access to adequate nutrition, which is essential for many patients receiving or awaiting treatment, was described as highly constrained. Similar research has shown that food insecurity, inadequate housing, and poverty are strongly associated with poorer health outcomes and reduced quality of life among patients with chronic illness and other vulnerable populations [38, 39, 40]. In the present study, the deterioration of living conditions emerged not as a separate social issue, but as a core part of the cancer care crisis itself.

### 4.3 Psycho‐Oncological Contribution and Survival‐Oriented Adaptation

Beyond documenting the disruption of oncology services, the findings advance current knowledge by showing how psycho‐oncological needs are produced through the interaction of treatment uncertainty, displacement, weakened support networks, and the inability to meet basic survival needs. Psycho‐oncology encompasses not only patients' emotional responses to cancer but also the broader psychological, social, and behavioural conditions that shape their illness experience and ability to engage with care [41]. The comparison between the pre‐war and wartime periods indicates that distress was not simply a response to cancer or war in isolation; rather, it intensified as fragile but partly functioning pathways for follow‐up, medication, referral, travel, and family assistance became inaccessible. This interpretation is consistent with evidence that conflict and humanitarian emergencies intensify psychological suffering while simultaneously weakening the health and social systems needed to respond to it (Inter‐Agency Standing Committee [42]; World Health Organization [43]. In this context, psychological support cannot be separated from practical access to food, transport, medication, shelter, symptom relief, and reliable information about treatment, because comprehensive care for people with life‐threatening illness requires attention to physical, psychological, social, and practical sources of suffering [43].

Participants' accounts also reveal how they navigated disruption: they delayed or stopped treatment when travel became unsafe, continued to pursue referrals despite closed crossings, rationed food or medical supplies, relied heavily on family members, and sought intermittent humanitarian assistance. These responses can be understood as survival‐oriented, constrained coping—adaptations made within severely restricted choices rather than evidence that patients could compensate for the collapse of care. The concept is used here as an interpretive description grounded in participants' accounts, rather than as a formal theoretical model. It highlights how agency in conflict settings may be expressed through efforts to preserve treatment access, manage scarce resources, and mobilize family or humanitarian support despite severe structural restrictions. This interpretation adds analytical depth to the descriptive themes by identifying a process through which material deprivation and treatment interruption shape fear, uncertainty, agency, and help‐seeking. It is also consistent with the stress‐buffering perspective, which proposes that social support can reduce some of the harmful effects of severe stress, although its protective capacity may be substantially weakened when families and communities are themselves exposed to displacement and deprivation [24].

The findings therefore extend the conflict‐oncology literature by specifying patient‐level psycho‐oncological priorities: predictable communication about treatment options, continuity of symptom and medication support, active navigation across referral pathways, sustained emotional support, and assistance with basic needs. Cancer patient navigation is specifically intended to identify and reduce barriers that prevent patients from obtaining timely and continuous care, particularly among populations experiencing social and economic disadvantage [44, 45]. The findings also demonstrate why psycho‐oncology in crisis settings requires an interdisciplinary model of care rather than stand‐alone counselling detached from patients' material and treatment realities. Such a model should integrate medical treatment and symptom management with nursing follow‐up, psychosocial assessment, care navigation, family support, and access to humanitarian resources. This approach is consistent with international guidance recommending integrated, person‐centred responses to physical, psychological, social, and practical suffering during humanitarian emergencies [42, 43].

### 4.4 Implications for Interdisciplinary Practice

The findings of this study have important implications for interdisciplinary practice across medicine, nursing, and social work. First, they show that cancer care in conflict settings cannot be understood solely in terms of diagnosis and treatment availability. Continuity of care is also shaped by displacement, poverty, transportation barriers, disrupted family support, psychological distress, and the inability to secure basic daily needs. Second, the findings suggest that effective responses for patients with cancer in war‐affected settings require integrated models of care that combine oncology services with psychosocial support, case management, referral coordination, nutritional support, and practical assistance.

From a nursing perspective, these findings highlight the importance of continuity of care, symptom monitoring, patient education, and supportive communication under highly constrained conditions. From a medical perspective, they underscore the need to preserve diagnostic and treatment pathways, medication access, and referral mechanisms for patients with life‐threatening conditions. From a social work perspective, the findings point to the importance of psychosocial assessment, family support, care navigation, advocacy, crisis intervention, and linkage to available humanitarian resources. The study therefore supports the view that cancer care in conflict settings must be approached as an interdisciplinary field of practice in which medical, nursing, and social work roles are complementary and mutually reinforcing.

Operationally, these integrated responses could be advanced through several coordinated mechanisms. Humanitarian organisations and local health actors could establish shelter‐ or community‐based lists of oncology patients, triage those requiring urgent medication, palliative care, diagnostic follow‐up, referral, or evacuation, and maintain priority referral pathways through available humanitarian coordination channels [21, 46, 47]. Mobile or community‐based oncology support teams could visit shelters and tent clusters to monitor symptoms, identify infection risks, support medication adherence, and document urgent needs for analgesics, chemotherapy‐related side‐effect management, ostomy supplies, nutrition, clean water, and transport assistance [46, 47].

More specifically, medical implications include the use of palliative care coordination protocols to prioritize severe symptom control, analgesia, urgent referral, and humanitarian evacuation when active oncology treatment is suspended [21, 43]. Nursing implications include mobile oncology nursing units or field symptom‐monitoring networks within shelters and tent clusters to identify fever, infection risk, chemotherapy‐related complications, wound or ostomy problems, nutritional deterioration, and medication interruption [46]; WHO Regional Office for the [47]. Social work implications include oncology patient navigation systems that map vulnerable patients, coordinate fast‐track access to nutrition, clean water, medication, transport, shelter, protection, and crisis intervention, and maintain communication between families, healthcare workers, humanitarian organisations, and referral actors [44, 45]; Inter‐Agency Standing Committee [42]. At the macro level, these actions require agreed referral forms, shared patient lists, focal points for oncology cases, and coordination between international humanitarian organisations, local health actors, and remaining healthcare workers [42, 47].

Where movement is unsafe or facilities are inaccessible, telehealth or telephone‐based support could provide remote psychosocial counselling, symptom follow‐up, and care navigation [37, 42, 48]. Interdisciplinary coordination can be implemented through brief case conferences, shared referral forms, or focal‐point systems linking physicians, nurses, social workers, mental health providers, and humanitarian actors [42, 46]. In this model, medical teams prioritize treatment continuity and palliative symptom control, nurses lead field symptom monitoring and patient education, and social workers coordinate patient navigation, psychosocial assessment, crisis intervention, family support, and linkage to food, water, shelter, medication, and protection services [21, 37, 42, 46].

### 4.5 Strengths and Limitations

This study has several strengths. It provides qualitative insight into the lived experiences of adults with cancer in the Gaza Strip under conditions of war and displacement, an area in which empirical evidence remains limited. It also captures experiences across both the pre‐war and wartime periods, allowing a more nuanced understanding of how pre‐existing vulnerabilities were intensified by conflict. In addition, the use of in‐depth interviews conducted by a research team member residing in the Gaza Strip supported context‐sensitive communication and facilitated the collection of rich and relevant accounts under highly constrained conditions.

At the same time, the composition of the purposive sample limits the breadth and transferability of the findings. Eleven of the 12 participants were women; consequently, the narratives predominantly represent women's experiences and cannot support conclusions about sex‐based differences or male‐specific psycho‐oncological needs. The account of the single male participant should not be regarded as representative of men living with cancer in the Gaza Strip. Future studies should seek larger samples with a more balanced representation of men and women to examine how gender roles, caregiving expectations, stigma, and help‐seeking may shape experiences of cancer care during conflict. Data collection under conditions of war and displacement may also have restricted access to potential participants and limited the range of experiences that could be documented.

The heterogeneity of cancer diagnoses, treatment requirements, and timing of diagnosis is another important limitation. The inclusion of several cancer types within a small sample prevented disease‐specific and treatment‐trajectory‐specific interpretation, including meaningful comparisons according to treatment modality, disease stage, prognosis, symptom burden, or diagnosis before versus during the war. The cross‐cutting themes should therefore not be interpreted as implying that experiences, needs, or treatment disruptions were equivalent across cancer diagnoses or treatment trajectories. Larger and more focussed studies, purposefully sampling participants according to cancer type, disease stage, or phase of treatment, are needed to identify diagnosis‐specific barriers and support needs.

Member checking was not feasible because participants were displaced, communication routes were unstable, and re‐contact could have increased participant burden or created safety concerns. Formal triangulation using additional qualitative data sources was also limited by the conditions of war. The analysis therefore relied primarily on interview data, team‐based review of coding and interpretation, and contextual clarification provided by the local field researcher. Humanitarian and health‐system reports were used to contextualise, rather than independently validate, the interpretation of participants' accounts.

The qualitative descriptive design was appropriate to the study aim because it enabled careful documentation of patient‐reported experiences and practice‐relevant needs in an under‐researched conflict setting. However, the study was not designed to generate a formal theory of coping or to examine the development of coping trajectories over time. The interpretation of survival‐oriented, constrained coping presented in the Discussion should therefore be understood as a provisional, empirically grounded analytic interpretation rather than as a developed or tested conceptual framework. Longitudinal and theory‐building research is needed to examine how care navigation, adaptation, and coping change over time and how they may differ according to sex, gender‐related circumstances, cancer diagnosis, and treatment trajectory.

## 5 Conclusion

This study highlights the complex and layered challenges faced by adults living with cancer in the Gaza Strip, both before and during the war. Even prior to the conflict, patients experienced substantial barriers related to healthcare access, treatment continuity, financial burden, and limited psychosocial support. With the onset of war, these challenges intensified dramatically, resulting in disrupted follow‐up, reduced access to medications and treatment, insufficient support mechanisms, and a profound deterioration in daily living conditions, including displacement and food insecurity. The findings demonstrate that cancer care in conflict settings is shaped not only by the disruption of health services, but also by broader social, economic, and humanitarian conditions that directly affect patients' capacity to survive, cope, and continue treatment. There is therefore an urgent need for integrated responses that combine medical care with psychosocial support, nutritional assistance, care coordination, and interdisciplinary collaboration across medicine, nursing, and social work. Such responses could be implemented through coordinated humanitarian referral pathways, mobile or community‐based oncology and nursing outreach, remote psychosocial support, and oncology patient navigation systems linking patients to treatment, palliative care, nutrition, clean water, medication, transportation, and crisis intervention. Importantly, the study advances psycho‐oncological understanding by demonstrating that distress and coping in conflict settings are inseparable from treatment uncertainty, disrupted support, and unmet basic survival needs. Patients' adaptations should therefore be understood as constrained responses to progressively diminishing options, rather than as evidence that they were able to compensate for the loss of continuous cancer care.

## Funding

The authors have nothing to report.

## Conflicts of Interest

The authors declare no conflicts of interest.

## Supporting information


Supporting Information S1

## Data Availability

The data that support the findings of this study are not publicly available due to ethical and privacy considerations related to the sensitive nature of the interviews and the vulnerability of the study population. De‐identified data may be available from the corresponding author upon reasonable request, subject to ethical approval and participant confidentiality requirements.

## References

[1] M. AlKhaldi , R. Kaloti , D. Shella , A. Al Basuoni , and H. Meghari , “Health System’s Response to the COVID‐19 Pandemic in Conflict Settings: Policy Reflections From Palestine,” Global Public Health 15, no. 8 (2020): 1244–1256, 10.1080/17441692.2020.1781914. 32552389

[2] S. Al Waheidi , “Breast Cancer in Gaza—A Public Health Priority in Search of Reliable Data,” ecancermedicalscience 13 (2019), 10.3332/ecancer.2019.964. PMC6834385 31921335

[3] WHO, Regional Office for the Eastern Mediterranean , “Gaza Patients’ Painful Journey to Cancer Treatment,” 2019, https://www.emro.who.int/opt/news/gaza‐patients‐painful‐journey‐to‐cancer‐treatment.html.

[4] A. N. Shamallakh and A. M. Imam , “Quality of Life in Patients With Cancer in the Gaza Strip: A Cross‐Sectional Study,” Lancet 390 (2017): S21, 10.1016/s0140-6736(17)32072-x.

[5] R. A. Bseiso and A. A. M. Thabet , “The Relationship Between Siege Stressors, Anxiety, and Depression Among Patients With Cancer in Gaza Strip,” Health Science Journal 11, no. 2 (2017): 499, 10.21767/1791-809X.1000499.

[6] R. S. Zeilani , W. Y. Younis , R. Albusoul , A. Hasanien , and A. H. Mansour , “The Buffering Role of Social Support on the Severity of Physical Symptoms Among Patients Living With Cancer,” International Journal of Palliative Nursing 29, no. 5 (2023): 204–215, 10.12968/ijpn.2023.29.5.204. 37224097

[7] W. Ammar‐Shehada , P. Bracke , and M. Ceuterick , “Experiences of Social Burden Amongst Survivors of Breast Cancer in Gaza: A Qualitative Study,” European Journal of Oncology Nursing 73 (2024): 102716, 10.1016/j.ejon.2024.102716. 39476734

[8] J. Leung , N. A. Pachana , and D. McLaughlin , “Social Support and Health‐Related Quality of Life in Women With Breast Cancer: A Longitudinal Study,” Psycho‐Oncology 23, no. 9 (2014): 1014–1020, 10.1002/pon.3523. 24700668

[9] B. Bortolato , T. N. Hyphantis , S. Valpione , et al., “Depression in Cancer: The Many Biobehavioral Pathways Driving Tumor Progression,” Cancer Treatment Reviews 52 (2017): 58–70, 10.1016/j.ctrv.2016.11.004. 27894012

[10] H. Abu‐Odah , A. Molassiotis , I. Y. Zhao , J. J. Su , and M. J. Allsop , “Psychological Distress and Associated Factors Among Palestinian Advanced Cancer Patients: A Cross‐Sectional Study,” Frontiers in Psychology 13 (2022): 1061327, 10.3389/fpsyg.2022.1061327. 36533049 PMC9755485

[11] UN , Gender Alert: Gaza—A War on Women’s Health (United Nations Office of the Special Representative of the Secretary‐General on Sexual Violence in Conflict, September, 2024).

[12] UNFPA , UNFPA Palestine Situation Report #11 – November 2024 (Covering Period: 01 September – 31 October 2024) (United Nations, 2024), https://www.un.org/unispal/document/unfpa‐sitrep‐01nov2024/.

[13] WHO Regional Office for the Eastern Mediterranean , “oPt Emergency Situation Update: Issue 68, 7 October 2023‐31 March 2026,” (2026), https://www.emro.who.int/images/stories/palestine/Sitrep_68.pdf.

[14] UNDP , “Gaza War: Expected Socioeconomic Impacts on the State of Palestine Update | May 2024,” (2024), https://www.undp.org/sites/g/files/zskgke326/files/2024‐05/2400257e‐gaza_war‐_expected_socioeconomic_impacts‐pb.pdf.

[15] A. J. Nashwan , “A Double Battle: Fighting Cancer in the Shadows of Conflict in Gaza,” Cureus 15, no. 11 (2023), 10.7759/cureus.48371. PMC10699498 38060746

[16] A. Tong , P. Sainsbury , and J. Craig , “Consolidated Criteria for Reporting Qualitative Research (COREQ): A 32‐Item Checklist for Interviews and Focus Groups,” International Journal for Quality in Health Care 19, no. 6 (2007): 349–357, 10.1093/intqhc/mzm042. 17872937

[17] Palestinian Ministry of Health , Infographics of Cancer Report 2022: Cancer Situation in Gaza Strip—Facts & Figures 2022 (Palestinian Health Information Center, Cancer Registry Center, 2023), https://www.moh.gov.ps/portal/wp‐content/uploads/2023/03/Infographics‐of‐Cancer‐Report‐2022‐English.pdf.

[18] S. Elo and H. Kyngäs , “The Qualitative Content Analysis Process,” Journal of Advanced Nursing 62, no. 1 (2008): 107–115, 10.1111/j.1365-2648.2007.04569.x. 18352969

[19] S. Massad , M. Isbeih , K. Abu Saman , et al., “Challenges of Access to Care for Children With Cancer Living in the Gaza Strip/Occupied Palestinian Territory in 2022: A Cross‐Sectional Study,” BMJ Global Health 11, no. 1 (2026): e015493, 10.1136/bmjgh-2024-015493. PMC12781985 41500662

[20] S. Mitwalli , W. Hammoudeh , R. Giacaman , and R. Harding , “Access to Advanced Cancer Care Services in the West Bank‐Occupied Palestinian Territory,” Frontiers Oncology 13 (2023): 1120783, 10.3389/fonc.2023.1120783. PMC10062449 37007067

[21] Y. Ahmed , “Enhancing Cancer Care amid Conflict: A Proposal for Optimizing Oncology Services During Wartime,” JCO Global Oncology 9 (2023): e2300304, 10.1200/go.23.00304. 38085039 PMC10846788

[22] M. Y. Abdullah , A. A. Alhayek , F. D. Alsuraihi , et al., “Cancer Palliative Care in Low‐Resource Settings: Challenges and Innovations Regeneration,” Journal of Healthcare Sciences 5, no. 7 (2025): 290–298, 10.52533/JOHS.2025.50707.

[23] S. Abuzerr and S. Najjar , “Cancer Treatment Challenges During Gaza's Humanitarian Crisis,” Eastern Mediterranean Health Journal 31, no. 2 (2025): 116–117, 10.26719/2025.31.2.116. 40116266

[24] S. Cohen and T. A. Wills , “Stress, Social Support, and the Buffering Hypothesis,” Psychological Bulletin 98, no. 2 (1985): 310–357, 10.1037/0033-2909.98.2.310. 3901065

[25] C. H. Kroenke , “A Conceptual Model of Social Networks and Mechanisms of Cancer Mortality, and Potential Strategies to Improve Survival,” Translational Behavioral Medicine 8, no. 4 (2018): 629–642, 10.1093/tbm/ibx061. 30016520 PMC6065533

[26] WHO , “World Mental Health Report: Transforming Mental Health for all,” (2022), https://www.who.int/publications/i/item/9789240049338.

[27] L. F. Higuita‐Gutiérrez , D. A. Estrada‐Mesa , and J. A. Cardona‐Arias , “Healthcare Inequities Experienced by Patients With Cancer: A Qualitative Study in Medellín, Colombia,” Patient Preference and Adherence 16 (2022): 1983–1997, 10.2147/ppa.s369628. 35958886 PMC9362511

[28] J. Zhao , N. Zhang , T. Hilal , J. M. Griffin , K. R. Yabroff , and N. Khera , “Health Insurance Literacy Among Patients Receiving Outpatient Cancer Treatment,” Cancer 130, no. 20 (2024): 3480–3486, 10.1002/cncr.35439. 39017818

[29] B. T. Allaire , D. Zabala , L. M. Lines , C. Williams , M. Halpern , and M. Mollica , “Associations Between Healthcare Costs and Care Experiences Among Older Adults With and Without Cancer,” Journal of Geriatric Oncology 14, no. 7 (2023): 101561, 10.1016/j.jgo.2023.101561. 37392562 PMC10527170

[30] S. S. Alrawa , E. S. Alfadul , M. M. A. Elhassan , and N. Hammad , “Five Months Into Conflict: Near Total Collapse of Cancer Services in Sudan,” ecancermedicalscience 17 (2023): ed128, 10.3332/ecancer.2023.ed128. 38414957 PMC10898907

[31] L. Basha , H. Ahmed , M. Hamze , et al., “Cancer and Syria in Conflict: A Systematic Review,” BMC Cancer 24, no. 1 (2024): 1537, 10.1186/s12885-024-13256-9. 39695449 PMC11656653

[32] A. Al‐Ibraheem , A. S. Abdlkadir , A. Mohamedkhair , et al., “Cancer Diagnosis in Areas of Conflict,” Frontiers Oncology 12 (2022): 1087476, 10.3389/fonc.2022.1087476. PMC9815758 36620568

[33] O. Shamieh , T. Kutluk , F. M. Fouad , R. Sullivan , and A. Mansour , “Cancer Care in Areas of Conflict,” Frontiers in Oncology 13 (2023): 1301552, 10.3389/fonc.2023.1301552. 38023225 PMC10646180

[34] A. J. Nguyen , M. E. Lasater , C. Lee , et al., “Psychosocial Support Interventions in the Context of Forced Displacement: A Systematic Review and Meta‐Analysis,” Journal of migration and health 7 (2023): 100168, 10.1016/j.jmh.2023.100168. 36816445 PMC9932448

[35] I. R. Bou‐Orm , M. Moussallem , J. Karam , et al., “Provision of Mental Health and Psychosocial Support Services to Health Workers and Community Members in conflict‐affected Northwest Syria: A Mixed‐Methods Study,” Conflict and Health 17, no. 1 (2023): 46, 10.1186/s13031-023-00547-4. 37794393 PMC10548701

[36] K. Dickson , S. Y. J. Ko , C. Nguyen , D. Minchenko , and M. Bangpan , “Mental Health and Psychosocial Support Programmes for Displaced Populations in Low‐and Middle‐Income Countries (LMICs): A Systematic Review of Process, Perspectives and Experiences,” Global mental health 11, (2024): e62, 10.1017/gmh.2024.56. 38774885 PMC11106547

[37] WHO , “Mental Health in Emergencies,” (2025), https://www.who.int/news‐room/fact‐sheets/detail/mental‐health‐in‐emergencies?utm_source.

[38] J. Hanmer , D. A. DeWalt , and S. A. Berkowitz , “Association Between Food Insecurity and Health‐Related Quality of Life: A Nationally Representative Survey,” Journal of General Internal Medicine 36, no. 6 (2021): 1638–1647, 10.1007/s11606-020-06492-9. 33409885 PMC8175545

[39] F. Gany , I. Melnic , J. Ramirez , et al., “The Association Between Housing and Food Insecurity Among Medically Underserved Cancer Patients,” Supportive Care in Cancer 29, no. 12 (2021): 7765–7774, 10.1007/s00520-021-06254-1. 34169329 PMC8225310

[40] V. Hubert , K. Phelan , L. I. Bozama , et al., “Food Insecurity, Feeding Practices and Associated Factors of Acute Malnutrition Among Children Under 5 Years of Age in a Post‐Conflict Context in the Kasai region, Democratic Republic of Congo: A Community‐Based Case‐Control Study,” BMC nutrition 11, no. 1 (2025): 106, 10.1186/s40795-025-01096-0. 40448235 PMC12123807

[41] J. C. Holland , “Psycho‐Oncology: Overview, Obstacles and Opportunities,” Psycho‐Oncology 27, no. 5 (2018): 1364–1376, 10.1002/pon.4692. 29749682

[42] Inter‐Agency Standing Committee , IASC Guidelines on Mental Health and Psychosocial Support in Emergency Settings (Inter‐Agency Standing Committee, 2007), https://interagencystandingcommittee.org/iasc‐task‐force‐mental‐health‐and‐psychosocial‐support‐emergency‐settings/iasc‐guidelines‐mental‐health‐and‐psychosocial‐support‐emergency‐settings‐2007. 10.1080/09540261.2022.2147420 36502397

[43] WHO , Integrating Palliative Care and Symptom Relief into the Response to Humanitarian Emergencies and Crises: A WHO Guide (World Health Organization, 2018), https://www.who.int/publications/i/item/9789241514460.

[44] H. P. Freeman and R. L. Rodriguez , “History and Principles of Patient Navigation,” supplement, Cancer 117, no. 15 Suppl (2011): 3539–3542, 10.1002/cncr.26262. 21780088 PMC4557777

[45] E. D. Paskett , J. P. Harrop , and K. J. Wells , “Patient Navigation: An Update on the State of the Science,” CA: A Cancer Journal for Clinicians 61, no. 4 (2011): 237–249, 10.3322/caac.20111. 21659419 PMC3623288

[46] F. J. Bausch , D. Beran , H. Hering , et al., “Operational Considerations for the Management of Non‐Communicable Diseases in Humanitarian Emergencies,” Conflict and Health 15, no. 1 (2021): 9, 10.1186/s13031-021-00345-w. 33632275 PMC7905755

[47] WHO Regional Office for the Eastern Mediterranean , Addressing Noncommunicable Diseases in Emergencies: A Regional Framework for Action (World Health Organization Regional Office for the Eastern Mediterranean, 2024), https://applications.emro.who.int/docs/NCDs‐emergencies‐eng.pdf.

[48] K. M. Shaffer , K. L. Turner , C. Siwik , et al., “Digital Health and Telehealth in Cancer Care: A Scoping Review of Reviews,” Lancet Digital Health 5, no. 5 (2023): e316–e327, 10.1016/S2589-7500(23)00049-3. 37100545 PMC10124999

