# Supplementary material for: Cancer Care in the Gaza Strip Before and During War: Patient Experiences of Treatment Disruption, Unmet Support Needs, and Interdisciplinary Implications for Medicine, Nursing, and Social Work
Source: Psychooncology. 2026 Aug 11;35(8):e70575. doi: 10.1002/pon.70575 (PMC13459516; doi:10.1002/pon.70575)
Supplement: Supplementary file 1 [file PON-35-e70575-s001.docx]

**Supplementary File 1. COREQ Checklist**

Manuscript title: Cancer care in the Gaza Strip before and during war: patient experiences of treatment disruption, unmet support needs, and interdisciplinary implications for medicine, nursing, and social work

Reporting guideline: Consolidated Criteria for Reporting Qualitative Research (COREQ): 32-item checklist for interviews and focus groups (Tong et al., 2007).

| **COREQ domain** | **Item** | **Checklist item** | **Location/response in manuscript** |
| --- | --- | --- | --- |
| Domain 1: Research team and reflexivity | 1 | Interviewer/facilitator | Reported in Methods: the Gaza Strip-based local field researcher conducted the interviews. |
|  | 2 | Credentials | Author affiliations and disciplinary backgrounds are listed on the title page; field researcher affiliation is stated in Methods. |
|  | 3 | Occupation | Reported through author affiliations on the title page and author roles in Methods. |
|  | 4 | Gender | Not reported in detail to avoid unnecessary identifying information; interviewer role is described. |
|  | 5 | Experience and training | Relevant disciplinary roles are reflected in author affiliations and team roles; interview guide was reviewed by experts. |
|  | 6 | Relationship established | Methods/Trustworthiness describe rapport establishment before interviews. |
|  | 7 | Participant knowledge of interviewer | Methods describe explanation of study purpose, voluntary participation, and confidentiality. |
|  | 8 | Interviewer characteristics | Methods/Trustworthiness describe local field researcher positionality and shared war context. |
| Domain 2: Study design | 9 | Methodological orientation and theory | Qualitative descriptive design and inductive qualitative content analysis are reported. |
|  | 10 | Sampling | Purposive sampling is reported in Setting and participants. |
|  | 11 | Method of approach | Participants were accessed in shelters, displacement centres, and tent settings through the local field researcher. |
|  | 12 | Sample size | Twelve adult cancer patients are reported. |
|  | 13 | Non-participation | Not systematically documented due to wartime fieldwork constraints. |
|  | 14 | Setting of data collection | Shelters, displacement centres, and tent settings in the Gaza Strip are described. |
|  | 15 | Presence of non-participants | Not systematically recorded due to displacement conditions; confidentiality procedures are described. |
|  | 16 | Description of sample | A de-identified participant characteristics table is provided. |
|  | 17 | Interview guide | Semi-structured interview guide topics are described in Methods. |
|  | 18 | Repeat interviews | No repeat interviews were conducted. |
|  | 19 | Audio/visual recording | Audio-recording with participant consent is reported. |
|  | 20 | Field notes | Contextual clarification by the local field researcher is described; formal qualitative field notes were not treated as a separate data source. |
|  | 21 | Duration | Interviews lasted approximately 45–60 minutes. |
|  | 22 | Data saturation | Data collection continued until sufficient depth had been reached to address the study questions. |
|  | 23 | Transcripts returned/member checking | Member checking was not feasible under active war and displacement conditions; this is acknowledged. |
| Domain 3: Analysis and findings | 24 | Number of data coders | More than one author reviewed interview materials and preliminary codes. |
|  | 25 | Description of coding tree | An illustrative coding tree is provided in Table 2. |
|  | 26 | Derivation of themes | Methods describe movement from meaning units to codes, categories, and analytical themes. |
|  | 27 | Software | No qualitative software is reported; analysis was conducted through team-based review and coding. |
|  | 28 | Participant checking | Member checking was not feasible; this is reported as a limitation. |
|  | 29 | Quotations presented | Direct quotations are presented with participant codes and contextual descriptors. |
|  | 30 | Data and findings consistent | Findings are supported by quotations within each theme. |
|  | 31 | Clarity of major themes | Six analytical themes are organized within two temporal categories. |
|  | 32 | Clarity of minor themes | Categories and themes are summarized in the results and illustrative coding table. |

Reference: Tong, A., Sainsbury, P., & Craig, J. (2007). Consolidated criteria for reporting qualitative research (COREQ): A 32-item checklist for interviews and focus groups. International Journal for Quality in Health Care, 19(6), 349–357. https://doi.org/10.1093/intqhc/mzm042
